# Random transposon insertion in the *Mycoplasma hominis* minimal genome

**DOI:** 10.1038/s41598-019-49919-y

**Published:** 2019-09-19

**Authors:** Fabien Rideau, Chloé Le Roy, Eveline Sagné, Hélène Renaudin, Sabine Pereyre, Birgit Henrich, Emilie Dordet-Frisoni, Christine Citti, Carole Lartigue, Cécile Bébéar

**Affiliations:** 1 University of Bordeaux, USC-EA3671 Mycoplasmal and Chlamydial Infections in Humans, Bordeaux, France; 2 INRA, USC-EA3671 Mycoplasmal and Chlamydial Infections in Humans, Bordeaux, France; 3 IHAP, Université de Toulouse, INRA, ENVT, Toulouse, France; 4 Institute of Medical Microbiology and Hospital Hygiene, Heinrich Heine University, Düsseldorf, Germany; 5 INRA, UMR 1332 de Biologie du Fruit et Pathologie, F-33140 Villenave d’Ornon, Gironde, France; 6 University of Bordeaux, UMR 1332 de Biologie du Fruit et Pathologie, F-33140 Villenave d’Ornon, Gironde, France

**Keywords:** Bacterial techniques and applications, Bacteriology, Bacterial genetics, DNA recombination

## Abstract

*Mycoplasma hominis* is an opportunistic human pathogen associated with genital and neonatal infections. Until this study, the lack of a reliable transformation method for the genetic manipulation of *M*. *hominis* hindered the investigation of the pathogenicity and the peculiar arginine-based metabolism of this bacterium. A genomic analysis of 20 different *M*. *hominis* strains revealed a number of putative restriction-modification systems in this species. Despite the presence of these systems, a reproducible polyethylene glycol (PEG)-mediated transformation protocol was successfully developed in this study for three different strains: two clinical isolates and the M132 reference strain. Transformants were generated by transposon mutagenesis with an efficiency of approximately 10^−9^ transformants/cell/µg plasmid and were shown to carry single or multiple mini-transposons randomly inserted within their genomes. One M132-mutant was observed to carry a single-copy transposon inserted within the gene encoding P75, a protein potentially involved in adhesion. However, no difference in adhesion was observed in cell-assays between this mutant and the M132 parent strain. Whole genome sequencing of mutants carrying multiple copies of the transposon further revealed the occurrence of genomic rearrangements. Overall, this is the first time that genetically modified strains of *M*. *hominis* have been obtained by random mutagenesis using a mini-transposon conferring resistance to tetracycline.

## Introduction

*Mycoplasma hominis* is an opportunistic pathogen that is a common commensal bacterium of the human female genital tract with the ability to cause genital and neo-natal infections and systemic infections in immunocompromised patients^1^. This pathogen is frequently associated with *Trichomonas vaginalis* and can influence gene expression in this parasite^2^. The genome of the reference strain *M*. *hominis* PG21 has been sequenced (665 kb, 27.1% GC)^3^ and is the second smallest genome known to be capable of sustaining life after that of *M*. *genitalium*^4^. An analysis of the genome sequence of this member of the class *Mollicutes* revealed that its ability to produce energy depends on the hydrolysis of arginine, which is in contrast to the glucose or urea used by most bacteria colonizing the same urogenital niche.

Our understanding of the metabolism and mechanisms of infection of this minimal organism remains severely limited, primarily because of the lack of genetic manipulation tools. A transformation method for this bacterium based on electroporation was described in 2000^5^. However, until the current study, the procedure could not be repeated. Another study describing the transfer of the Tn916 transposon from *Streptococcus faecalis* to *M*. *hominis* has been reported^6^, but these results could not be reproduced either. More recently, a technique combining random chemical mutagenesis and high-throughput screening for nucleotide polymorphisms has been used in *M*. *hominis* PG21^7^. This reverse genetic method, called TILLING, for Targeting-Induced Local Lesions IN Genomes, allowed the first *M*. *hominis* PG21 mutants to be produced. Although this achievement paved the way for the development of functional genomics in *M*. *hominis*, the process remains rather fastidious for routine application and may generate multiple undesired mutations across the genome^7^.

A synthetic biology approach is now commonly used for mycoplasma species belonging to the mycoides cluster^8^. In this procedure, the mycoplasma genome is first isolated from a bacterial cell and is subsequently cloned into the yeast *Saccharomyces cerevisiae*, where it can be modified at large scale using the genetic tools available for this host, such as the tandem repeat coupled with endonuclease cleavage (TREC) or the CRISPR/Cas9 systems^9,10^. Next, the highly modified genome is transplanted into a recipient cell of the same species or a phylogenetically proximate species^11^ to generate the cognate mutants. This technique is currently under development for *M*. *hominis*, but the last transplantation step is yet to be successfully achieved^12^. As with any transformation procedure^13^, the restriction-modification (R-M) barrier must be taken into consideration in the genome transplantation process^14^. Indeed, incorrectly methylated genomes could be immediately hydrolyzed by the restriction enzymes present in the recipient cells^15^.

Over the past decades, efforts have been made to develop protocols for DNA transfer in *M*. *homini*s with no conclusive results. However, such procedures were successfully generated for both *M*. *arthritidis*, the closest relative of *M*. *hominis*^16^, and for *Ureaplasma parvum*, a human pathogen known to be difficult to transform, that shares the same ecological niche as *M*. *hominis*^13^. These results encouraged us to pursue the development of a transformation procedure for *M*. *hominis*. In this study, we report a reproducible polyethylene-glycol (PEG)-based transformation protocol for *M*. *hominis* using plasmids carrying a transposon.

## Results

### Restriction-modification system analyses

Many restriction-modification systems (R-M systems) have been described in the genomes of mollicutes species (Supplementary Fig. S1), with several having been functionally characterized^8,16–18^. These systems provide immunity against foreign DNA and may therefore constitute an important barrier to both natural and artificial bacterial transformations^14,19^. Thus, to facilitate the development of a transformation procedure for *M*. *hominis*, we sought a strain containing the fewest of these defense mechanisms.

Twenty sequenced *M*. *hominis* genomes (13 from clinical strains described in this study, six already published^3,20–22^, and the M132 reference strain) were analyzed and compared using Molligen and Rebase databases^23,24^. Many putative R-M systems were identified (Table 1), with each strain studied containing between two and six complete R-M systems. Interestingly, two putative R-M systems were shared by all strains, the type I EcoR124II-like system and a type III system. The type II Sau96I-like system is present in 19 out 20 strains, but 7 seem to harbor an incomplete system. Other predicted systems were rather diverse in terms of their type and specificity (three different type I and seven different type II systems), and/or copy numbers (four copies of an incomplete type II DpnII-like system were predicted in the strain 3364) (Table 1). R-M systems were considered to be incomplete if one or more subunits were missing.

**Table 1:** *In silico* analyses of restriction-modification systems in the sequenced *M*. *hominis*.

*M*. *hominis* strains^a^	Type I			Type II							Type III	Type IV	Total
	EcoR124II	EcoKI	BcgIA	Sau96I	HaeIII	FokI	DpnII	Eco57I	BspRI	VspI		McrBC	
*PG21*	♦○^b^			○			♦○				♦		6
*ATCC 27545*	♦○			♦							♦		4
*ATCC 33131*	♦			♦○							♦		4
*AF1*	♦♦			♦○			♦			♦	♦		7
*AF3*	♦		○	♦○			♦				♦		6
*PL5*	♦	♦	○	○○			♦		○		♦		8
**M132** ^**c**^	♦	♦	○	○			♦				♦	♦	7
35	♦			♦♦○		○					♦		6
331	♦	♦		♦♦		○	○○○				♦		9
2674	♦		○	♦♦			○				♦♦		7
3299	♦○				○		○○○				♦		7
3364	♦	♦		○			○○○○				♦	○	9
3631	♦		○	○			○				♦		5
**4016**	♦♦			♦○○		○					♦		7
4235	♦		♦	○							♦		4
4788	♦			○			○	○			♦		5
4796	♦○		○	♦○○		○					♦		8
**5012**	♦		○	♦○		○	○○				♦		8
5060	♦		○	♦		○	♦○				♦		7
5096	♦○		○	♦○			○				♦		7

^a^Strains in italic correspond to strains with a genome sequence available in the NCBI database.

^b ♦^Corresponds to one complete copy. ^○^Indicates a putative incomplete system.

^c^Strains in bold are transformable strains.

This analysis suggested a great diversity of R-M systems in the *M*. *hominis* lineage. Since no strain had a very low number or no R-M systems, we decided to pursue further investigations with the strains available in the laboratory: two reference strains (PG21 and M132) and the 13 clinical isolates (referred to as 35, 331, 2674, 3299, 3364, 3631, 4016, 4235, 4788, 4796, 5012, 5060, and 5096).

### Development of the *M*. *hominis* PEG-mediated transformation protocol

The PEG-mediated transformation protocol for *M*. *hominis* was developed based on the previously developed transformation protocols for *M*. *arthritidis*^16^ and *U*. *parvum*^13^.

**Figure 1:**
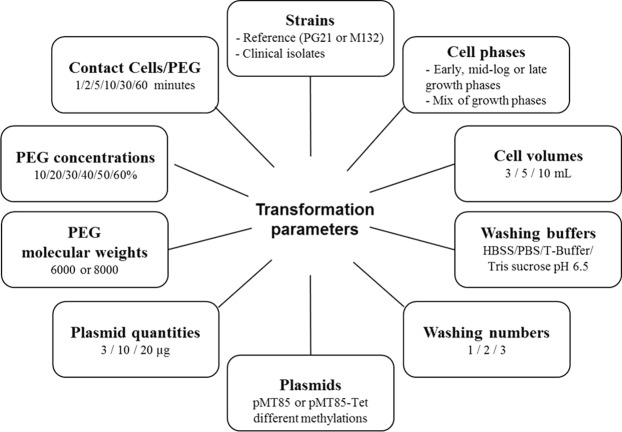
Variable parameters during *M*. *hominis* transformation assays. All of the different conditions assayed during the development of the transformation protocol are detailed in rectangles.

Many different transformation conditions were tested using the plasmid pMT85-Tet^25^ to generate genetically modified *M*. *hominis* mutants. The first assays were performed using the reference strain PG21, but no transformants were obtained. Subsequently, we attempted a series of experiments using the *M*. *hominis* reference strain M132. Two transformants were produced using a preliminary protocol, after which efforts were made to improve the reproducibility and efficiency of the protocol. In particular, we optimized conditions for *M*. *hominis* cell growth, the wash buffer composition, the methylation of the plasmid to be introduced and the PEG concentration, molecular weight and time of contact with the cells. Close to 150 variations of the protocols were tested, and the primary parameters that were adjusted are summarized in Fig. 1.

The impact of *M*. *hominis* M132 growth phase on the transformation protocol was evaluated with respect to its growth over time. Three culture phases (early, mid-log and late) were defined and used separately or in combination for transformation assays (Supplementary Fig. S2). The best efficiencies were obtained using a mix of the three growth phases compared to a particular growth phase.

Different wash buffers, which were also used to prepare the PEG solution, were tested during the protocol optimization. We tested PBS and Tris-sucrose (pH 6.5), two buffers commonly used for mycoplasma transformations, the eukaryotic cell culture buffer HBSS, and the T-Buffer used to transform *M*. *arthritidis*. While no transformants were generated using the Tris-sucrose (pH 6.5) and HBSS buffers, some transformants were obtained using PBS and T Buffer (Tris 10 mM, pH 6.5). However, the results obtained using PBS buffer were not reproducible.

Another important parameter considered was the methylation of the plasmid pMT85-Tet, because, as previously mentioned, R-M systems may have significant impact on transformation efficiency. The attempted transformation of *M*. *hominis* using unmethylated plasmid yielded no colonies. To protect the plasmid against restriction endonucleases and develop a protocol that could be broadly applied, we attempted to identify enzymes that could methylate the plasmid DNA at multiple sites. We tested two different commercial methyltransferases, the methyltransferase SssI, which methylates CpG islets, and the methyltransferase CviPI, which methylates GpC islets. In our experiments, transformants were only obtained using plasmid that had been methylated with the CpG methyltransferase. The methylation of pMT85-Tet with both the SssI and CviPI methyltransferases did not increase the transformation efficiency. Here, we did not address the adenine methylation issue mainly because we noticed that (1) most of these systems are either incomplete or absent in the strains of interest (Tables 1 and S1) and (2) some systems (as the GATC restriction-modification system) are present in the DH5α *E*. *coli* strain used to propagate the plasmid. This means that methyl groups are added to the adenine of the sequence 5′-*GATC-3*′ by the *E*. *coli* dam methylase prior transformation. Fully protected before the entry of the plasmid in *M*. *hominis* cells, the plasmid cannot be cleaved at those specific sites by the *M*. *hominis* cognate restriction enzyme.

With respect to PEG-mediated protocol, the initial transformation assays were performed using 40 to 50% PEG 8000, a concentration range that is commonly used for *M*. *arthritidis* and *U*. *parvum*. Assays using PEG of different molecular weights and concentrations were also tested. We observed that the concentration of PEG used was crucial to the success of the transformation. Transformations using low concentrations of PEG (10, 20, and 30%) were unsuccessful, while those using high concentrations (40, 50, and 60%) yielded a total of 24 transformants on selective media. The best efficiency was observed using 60% PEG, but the results were not reproducible. Only 40% PEG based protocol gave reproducible results (Table 2). Finally, different times of contact between cells and PEG were investigated (1, 2, 5, 10, 30, and 60 min). The most transformants were obtained when cells were incubated for 30 min with the methylated plasmid in presence of 40% PEG 8000 (Table 2). This table focuses only on the conditions that yielded transformants. The transformation efficiency average from at least three independent experiments is shown as well as the minimal and maximal transformation efficiency obtained in each condition. Several other parameters were investigated, including the culture volume (3 to 10 mL), the number of washes (1 to 3) and the quantity of plasmid (1 to 20 µg) (Fig. 1), but their impact on transformation efficiency was unclear.

**Table 2:** Successful transformation parameters and transformation efficiencies (in number of transformants/CFU/µg of plasmid) for *M*. *hominis* PEG-mediated transformation.

Transformation conditions			Number of successful repeats/Number of attempted experiments*	Transformation efficiency average [min-max]
PEG concentration (%)	Cell/PEG time of contact (minutes)	Washing buffer		
40	10	T-Buffer	3/6	4.43 × 10^−10^ [1.1 × 10^−10^ − 8.7 × 10^−10^]
40	30	T-Buffer	6/7	7.6 × 10^−10^ [1.8 × 10^−10^ − 2.2 × 10^−9^]
40	60	T-Buffer	1/3	2.9 × 10^−10^
50	10	PBS	2/5	6.45 × 10^−10^ [6.1 × 10^−10^ − 6.8 × 10^−10^]
50	30	PBS	1/3	6.8 × 10^−10^
50	60	PBS	1/3	2.7 × 10^−10^
60	30	PBS	1/3	2.3 × 10^−9^

^*^The results of 30 experiments are shown here. Many others have been attempted but yielded no transformants. We chose not to show them and focus on the conditions that led the apparition of transformants.

The results of these experiments led to the development of a reproducible PEG-mediated transformation protocol (see Material and Methods) that yielded M132 transformants at an efficiency of about 10^−10^ transformants/cell/µg of plasmid, with the highest score reaching 2.3 × 10^−9^ transformants/cell/µg of plasmid (Table 2).

The protocol was tested using 13 other clinical strains available in the laboratory that were susceptible to tetracycline, with only transformants obtained for strains 4016 (three mutants) and 5012 (one mutant).

### Genotypic analyses of *M*. *hominis* transformants

Colonies generally appeared on selective plates after 3 to 14 days of incubation, with most colonies exhibiting a typical “fried-egg” morphology that is characteristic of the wild-type *M*. *hominis* strain.

All of the transformants tested were positive by PCR for the *tet*(M) gene, suggesting that the transposon in the pMT85-Tet plasmid was present into the *M*. *hominis* cells. Moreover, the transformants were confirmed as *M*. *hominis via* sequencing of the 16S rDNA PCR products.

Further analyses were performed to precisely identify the site of transposon insertion. Single-primer PCRs using the gDNA of 24 transformants was followed by Sanger sequencing of the PCR products, with results obtained for 16 of the 24 mutants. For these 16 mutants, the sequencing data showed that only one copy of the transposon was present in the bacterial genome and that its insertion seemed to occur at random position in the genome (Table 3). Transposons were found inserted into intergenic regions, a hypothetical protein, lipoprotein-encoding genes, or at the 3′ end of essential genes (Table 3). For the remaining eight isolates, insertion sites for two could not be determined because of technical problems, while six had sequencing profiles showing several superimposed peaks, suggesting that several copies of the transposon were present in the genome (see below).

**Table 3:** Position of the *tet*(M) gene in the *M*. *hominis* M132 transformants.

*M*. *hominis* transformant	*tet*(M) position in the genome	Gene mnemonic	Gene product	Gene length (bp)	*tet*(M) position in the gene
M132-21	384,966	Mhom132_03040	Tyrosine recombinase XerC	1,035	471
22-1	384,682	Mhom132_03040	Tyrosine recombinase XerC	1,035	187
22-4	598,354	Mhom132_05080	tRNA-specific adenosine deaminase TadA	477	438
22-5	684,900	Mhom132_06040	dUTPase	528	21
28-2	297,373	Mhom132_02390	Putative P75 precursor	1,971	846
28-3	574,159	Mhom132_04900	Lmp-related protein	5,394	276
34-1	449,450	—	Non-coding region	—	—
35-4	699,164	Mhom132_06190	Restriction enzyme BcgIA subunit alpha	2,340	2,221
35-11	635,100	Mhom132_05560	Efflux magnesium and cobalt protein CorC	1,305	758
36-1	539,928	Mhom132_04540	Hypothetical Protein	783	104
37-1	71,023	—	Non-coding region	—	—
39-4	689,881	Mhom4016_05850	P120’ protein	2,748	842
39-5	290,526	Mhom4016_02610	Lmp-related protein	1,899	429
41-1	278,839	Mhom132_02270	ABC transporter	1,824	1,117
43-1	130,814	Mhom5012_01160	Cell division protein FtsZ	1,146	943
46-2	448,380	—	Non-coding region	—	—

### Phenotypic analyses of *M*. *hominis* mutant 28-2

**Figure 2:**
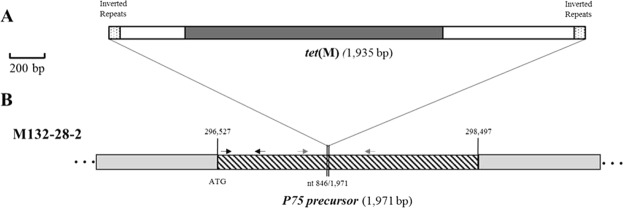
Insertion site of the *tet*(M) gene in the genome of the *M*. *hominis* M132 transformant 28-2. (**A**) Scheme of the transposon inserted into the *M*. *hominis* M132 genome. The region contains inverted repeats (point rectangles), the sequence of the pMT85 plasmid (white rectangles) and the *tet*(M) gene (dark gray rectangle). (**B**) Scheme of insertion site for transformant 28-2. Hatched rectangles represent the *M*. *hominis* M132 gene where the *tet*(M) gene inserted. Double thin lines represent the position of the insertion inside the *M*. *hominis* M132 gene. Clear gray rectangles correspond to sequence of the bacterial genome. Numbers above single thin lines indicate the position around the genome. Black arrows indicate the position of the PCR primers P75-F1 and P75-R1, and gray arrows indicate the position of the PCR primers P75-F2 and P75-R2.

The *M*. *hominis* mutant 28-2 was of particular interest, as the *tet*(M) gene was integrated in the middle of a gene encoding a precursor of the P75 lipoprotein that is potentially involved in the pathogenicity of *M*. *hominis* (Fig. 2). The expression of the gene encoding P75 was investigated by semi-quantitative RT-PCR. A small transcript corresponding to the beginning of the gene (before the *tet*(M) insertion) could be detected, whereas no transcript was produced around the insertion site of the transposon, suggesting that only a short transcript was produced for the gene (Supplementary Fig. S3).

**Figure 3:**
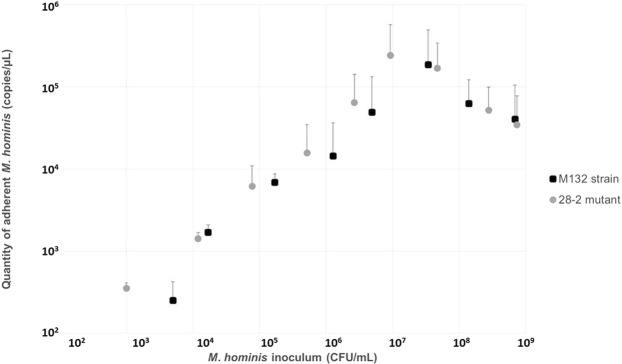
Test of adhesion to HeLa cells. The graph shows the quantity of adhered *M*. *hominis* (copies/µL) as a function of the *M*. *hominis* inoculum (in CFU/mL). Blue points correspond to the M132 wild-type strain and gray points correspond to the 28-2 mutant. This experiment was performed in triplicate, and average values are represented. Standard deviation represents the above points by vertical lines.

The ability of this mutant to adhere to HeLa cells was tested together with the *M*. *hominis* M132 wild-type strain. No difference in adhesion was observed between the M132 wild-type strain and the 28-2 mutant, suggesting the mutant fully retained its ability to adhere to eukaryotic cells. For both samples, the quantity of adhered *M*. *hominis* increased linearly as a function of the inoculum (Fig. 3). As expected, the correlation disappeared at high concentrations of *M*. *hominis* cells.

### Detection of large DNA rearrangements by WGS

**Figure 4:**
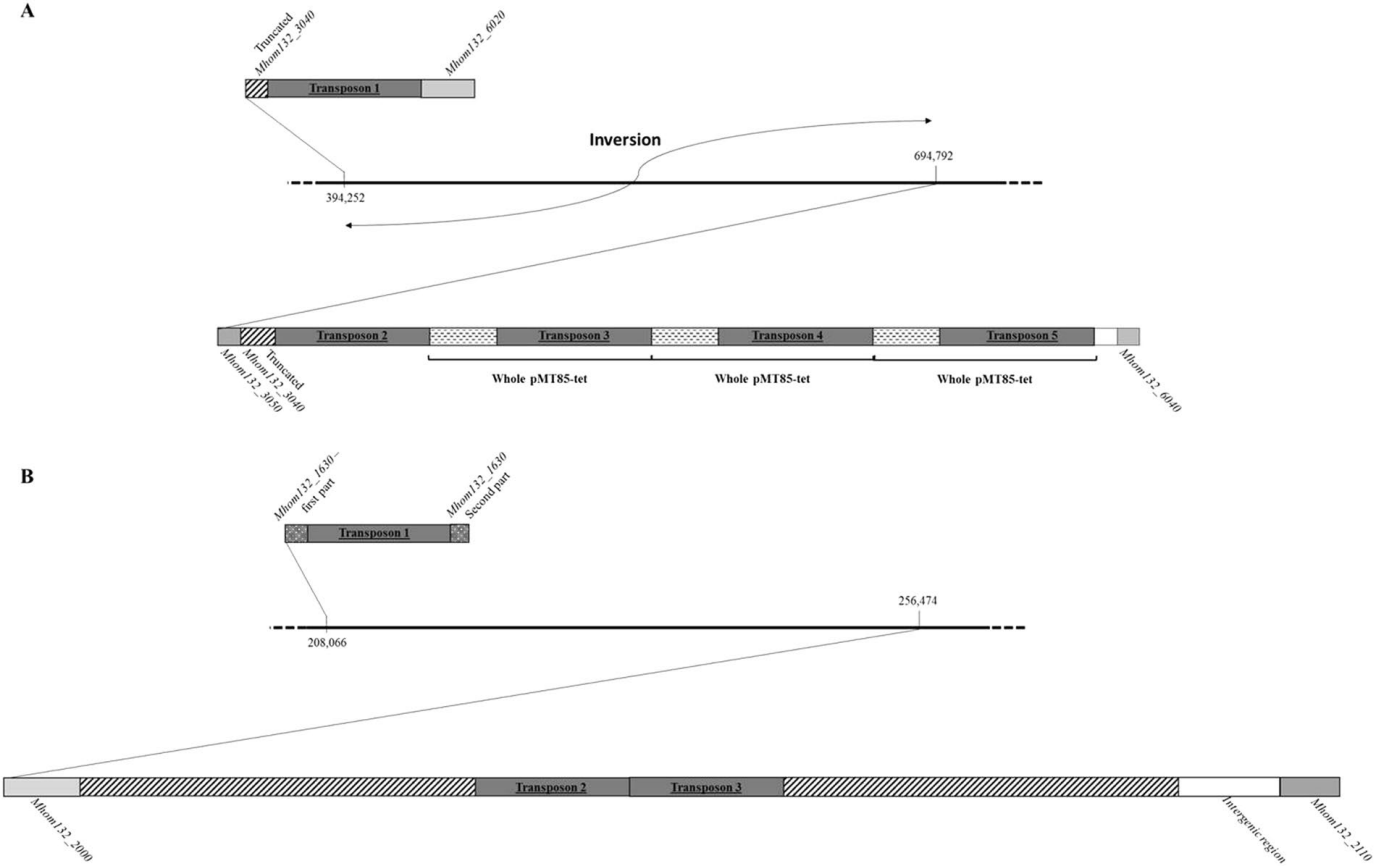
Transposon insertions in the mutants 28-1 **(A)** and 29-1 (**B**). Black lines correspond to bacterial genome portions. The numbers indicate the insertion position of *tet*(M). Hatched rectangles in (**B**) represent the duplicated region.

To confirm that the genomes of some mutants carried multiple copies of the transposon, the genomes of two mutants (28-1 and 29-1) were completely sequenced. The WGS data revealed the presence of variable copy numbers of the transposon throughout the genomes *M*. *hominis* of the two assayed clones. Indeed, five copies of the transposon were identified in the genome of transformant 28-1, while three copies were identified in the genome of transformant 29-1. Among the five transposon copies present in transformant 28-1, four were part of tandem repeats, three of which included copies of the entire pMT85-Tet plasmid (Fig. 4), while the remaining copy of the transposon was located 300 kb away. Surprisingly, this 300-kb region flanked by transposons was inverted compared to the wild-type strain. This inversion resulted in the disruption of the tyrosine recombinase-encoding *xer*C gene into two sections. For clone 29-1, a perfect duplication of approximately 9 kb flanked the double insertion of *tet*(M) at position 256,474 of the genome (Fig. 4). The third copy was observed to be inserted within a conserved hypothetical protein-encoding gene at position 208,266 of the genome. Altogether, these results suggested that large DNA rearrangement events in the *M*. *hominis* genome occurred adjacent to insertions of multiple copies of the transposon.

### Analyses of the antibiotic resistance of clones carrying several copies of the *tet*(M) gene

Minimal inhibitory concentrations (MICs) to tetracycline were determined to assess whether the copy number of the *tet*(M) gene influenced the level of tetracycline resistance. Compared to M132, whose MIC for tetracycline was 0.125 µg/mL, the 28-2 mutant, which carries only one copy of *tet*(M) gene, had an MIC of 8 µg/mL. This value was similar to that of the 29-1 mutant (16 µg/mL), which had three copies of the transposon. Interestingly, the clone 28-1, harboring five insertions of the *tet*(M) gene, had an MIC for tetracycline that was two to four times higher than that of the other mutants (32 µg/mL).

## Discussion

In this study, we developed a reproducible PEG-based transformation protocol for *M*. *hominis*. We showed that the procedure can be used for random mutagenesis using a plasmid carrying a mini-transposon. In the future, we believe that the use of this procedure could lead to a protocol for the directed mutagenesis of *M*. *hominis*, which has been accomplished for other mycoplasma species^26^.

To achieve this goal, we first sought to identify an *M*. *hominis* strain containing a low number of R-M systems. These systems are composed of a restriction endonuclease and a DNA methyltransferase, the latter of which prevents DNA cleavage by the cognate endonuclease (except for the type IV R-M system), and are well known to be a barrier to DNA transformation^14,27^. The genomes of 20 *M*. *hominis* strains were scrutinized for the presence of such systems, with the *in silico* analysis predicting the presence of numerous R-M systems encoded within the assayed strains (Table 1 and Supplementary Table S1). All of the studied genomes harbored a minimum of four systems total (complete plus incomplete), with up to nine systems identified for some clinical isolates (331 and 3364). Although no information is available regarding their activity, the high prevalence of R-M systems in the assayed *M*. *hominis* genomes combined with their high diversity (types I, II, III and potentially IV) may explain the difficulties encountered by the research community in developing strategies to genetically modify *M*. *hominis*.

Regarding the transformation protocol, several parameters appeared to be crucial to be successful (Fig. 1). First, we observed that the success of transformation depended on the selected strains. The PG21 strain was initially selected for our experiments as the most widely studied *M*. *hominis* representative^7,12^. Unfortunately, because no satisfying results were obtained, our efforts were redirected toward other strains. Among the 15 other strains tested, only three (M132, 4016 and 5012) were successfully transformed, although the reason for the observed success using these strains could not be ascertained. Indeed, the genomes of those three strains contained 5, 4 (7 including incomplete copies) and 3 (8 including incomplete copies) different complete R-M systems respectively; that is to say, a similar number of R-M systems as most of the other strains assayed (Table 1 and Supplementary Table S1). Moreover, all three strains contained different types of R-M systems (types I, II, III), sometimes in duplicate or triplicate (clinical strains 4016 and 5012). Although some R-M systems are perhaps not active, this is certainly not all of the systems detected, mainly because, bacteria that are subjected to degenerative evolution, such as mycoplasmas, have a tendency to lose genetic material that is not essential for their survival. Thus, may all these observations suggest that barriers other than R-M systems, not yet identified, may exist and prevent artificial DNA transfer in *M*. *hominis*?

It should be pointed out that a potential type IV R-M system (members of which are only composed of a restriction enzyme that cuts methylated DNA) was predicted in the *M*. *hominis* M132 genome. This system shares similarities with the *E*. *coli* McrBC system, which cleaves DNA containing methylcytosine on one or both strands^28^. In our assays, successful transformation uniquely occurred when the plasmid DNA was treated with the CpG methyltransferase SssI, suggesting that the McrBC-like system is present but either not functional under our experimental conditions or not active in the provided substrate. In any case, all assays performed with unmethylated plasmid or plasmid methylated with the GpC methyltransferase CviPI did not yield any transformants.

Another important parameter to consider was the growth phase of the bacteria used in transformation assays. Indeed, transformants were only obtained when an equal volume of the early, mid and late log phases were combined (Supplementary Fig. S2). Although this result was reported for *U*. *parvum*^13^, early or mid-log phase bacterial cultures are generally preferred in many other well-established transformation protocols^29–32^. Together with the bacterial growth phase, we noticed that some wash buffers were more suited to the transformation of *M*. *hominis* species than others. The T-Buffer used in the *M*. *arthritidis* transformation^16^ was successful, whereas other buffers commonly used for mycoplasmas (PBS or 10 mM Tris-HCl and 0.5 M sucrose, pH 6.5) were not.

The last crucial parameters to optimize for the *M*. *hominis* transformation procedure were the molecular weight and the concentration of the fusogenic chemical agent (PEG) used for transformation. The time of contact between the bacterial cells and the plasmid mixture was also of high importance. The optimal procedure developed required the use of PEG with an average molecular weight of 8,000 g/mol (PEG 8000) at high concentrations (40%, 50%, or 60% in T-Buffer), where the time of contact with bacterial cells/plasmid mixture lasted for 10 min or more. This need for the latter adjustment was unexpected, as most of the previously published PEG-mediated transformation protocols recommended that the incubation time of contact not be allowed to proceed for more than 2 min due to the presumed toxicity of the PEG toward the cells. In our experiments, we did not observe a higher mortality of *M*. *hominis* cells when their membranes were permeabilized by PEG for 10 to 30 min compared to 2 min (data not shown). Finally, for the transformation experiment to succeed, we noticed that more than 10 µg of plasmidic DNA are required since lower quantity did not yield any transformants.

Two antibiotic resistance-encoding genes were tested for selection during the transformation experiments. In contrast to the tetracycline determinant, the gentamicin resistance gene only allowed for the recovery of false-positive clones corresponding to spontaneous resistance mutants (data not shown). The development of a replicative plasmid containing the origin of replication of *M*. *hominis* was attempted, although convincing results were not obtained (data not shown).

By combining all of these parameters, we generated a reliable transformation protocol for *M*. *hominis*. Relatively low efficiencies were obtained for each experiment (up to 2.3.10^−9^ transformants/cell/µg of plasmid (corresponding to 1 to 3 transformants per experiment), indicating that the protocol should be further optimized. However, the results obtained using this protocol are reproducible and resulted in the generation of genetically modified *M*. *hominis* cells by transposon mutagenesis. The protocol may be further refined by counteracting of R-M systems. For example, the transformed DNA could be protected by *in vitro* methylation before its entry into the cells. In the current protocol, plasmids are methylated with the commercial methyltransferase SssI, which only provides protection against some restriction endonucleases (those recognizing CpG sites), but not all. DNA protection with other types of cytosine methyltransferases, but also with adenine methyltransferases should be considered, both type of R-M systems being found in *M*. *hominis* genomes (Tables 1 and S1). The best way to tackle the problem would be to prepare *M*. *hominis* cellular crude extracts (or *M*. *hominis* recombinant methyltransferases) in order to be able to methylate the exogenous DNA with *M*. *hominis* endogenous methyltransferases just before transformation^8^. However, it could be laborious and time-consuming to obtain crude extracts with fully functional methyltransferases or determine the laboratory conditions in which recombinant methyltransferases are active. The incoming DNA can be degrading by R-M systems once in the cell, but can also be degraded by surface nucleases before its entry into the cell^33^. Most nucleases require a divalent cation as a cofactor to be fully active (usually Mg^2+^ or Ca^2+^). Washing the cells with the chelators EDTA or EGTA may help neutralizing their activity and increasing the number of transformants. Finally, reaching better transformation efficiencies may also rely on the construction of a plasmid that is more adapted to *M*. *hominis*. Different promotors for driving the *tet*(M) expression gene could be tested, and a codon optimized version of the *tet*(M) gene could also be designed.

Mutants of interest were isolated over the course of our transformation experiments, such as mutant 28-2, which harbors the *tet*(M)-carrying transposon within the P75 lipoprotein gene that is potentially involved in the cytoadherence of *M*. *hominis*^34,35^. After verifying that the gene was knocked-out by PCR and RT-PCR, the adhesion ability of the mutant strain was tested using HeLa cells (Fig. 3**)**. The results showed no difference in adhesion between the M132 wild-type strain and the derivative mutant 28-2. A few hypotheses should be considered with respect to this result: (i) the truncated protein may still be expressed at the surface with a conformation allowing adhesion to HeLa cells, antibodies against the P75 protein could be helpful to confirm this hypothesis by Western Blot; (ii) the adhesion test to HeLa cells was not sensitive enough, a possibility for which a negative control using a nonadherent strain (which is not currently available) would allow us to confirm or rule out this hypothesis; and, (iii) the absence of one protein is not sufficient to observe an altered adhesion, as other surface proteins have been shown to be important in *M*. *hominis* adhesion^36,37^. Additionally, adhesion experiments using other cell lines could certainly be done, but those tests would require some optimizations.

The use of next generation sequencing for deciphering the genome sequence of two transformants (mutants 28-1 and 29-1), confirmed the presence of multiple copies of the transposon throughout their genomes. A large 300-kb genomic inversion was observed in mutant 28-1, which caused the inactivation of a gene annotated as putative tyrosine recombinase XerC. This type of recombinases is known for its role as a mediator in site-specific recombination events and inversion events in bacteria^38–43^. Interestingly, the transposon was shown to be integrated into this gene in three mutants (two with a single insertion and one with a multiple insertion) out of the 24 obtained during this study. We concluded that *xerC* was certainly a “hotspot” of integration of this transposon in *M*. *hominis* M132 species, similarly to the four genes MG339 (recA), MG414 (Hypothetical protein), MG415 (hypothetical protein), and MG428 (putative regulatory protein) in *M*. *genitalium*. which constituted about 31% of the total transposon insertions during gene essentiality study^44^. We could also hypothesize that mutations in this locus facilitate fitness and growth. Multiple insertions of transposons as well as whole plasmid integration have been previously observed in mycoplasmas using the transposons Tn4001 and Tn916^13,45–50^. In the pMT85-Tet plasmid, the transposase-encoding gene was moved from the transposon to limit multiple insertions and full plasmid integration^25,45^. To the best of our knowledge, we showed for the first time the presence of multiple transposon integrations, whole plasmid recombination and highlighted large DNA rearrangements in the genomes of *M*. *hominis* cells that were certainly induced by the presence of the plasmid. It would be interesting to analyze more mutants with suspected multiple insertions (i) to look for the presence of small or large genomic rearrangements, (ii) study their nature (insertions, duplications, inversions) and finally (iii) to understand the underlying mechanisms.

Another interesting outcome of this work was that the *tet*(M) copy number may influence the level resistance of a strain to an antibiotic. In our experiments, clone 28-1, which had the highest copy number of the *tet*(M) gene, has a tetracycline MIC that is two to four times higher than that observed of the other mutants (32 µg/mL).

Altogether, these data demonstrate that we are now capable of obtaining some genetically modified *M*. *hominis* mutants by random mutagenesis. Even though the transformation efficiency obtained using this protocol is currently low, these results are encouraging and lay the foundation for the function studies of genes in this bacterium. These results may also pave the way toward the development of genome transplantation to permit targeted mutagenesis and direct inactivation of genes of interest in *M*. *hominis*.

## Materials and Methods

### Mycoplasmas strains, culturing and numeration

The *M*. *hominis* reference strains PG21 (ATCC 23114) and M132 (ATCC 43521) and the clinical isolates 2674, 3299, 331, 3364, 35, 3631, 4016, 4235, 4788, 4796, 5012, 5060, and 5096 (SRA accession number: PRJNA493181) were grown at 37 °C in Hayflick modified medium supplemented with arginine for 24 to 48 hours^51^. Transformants were cultured in the same medium containing 2 µg/mL of tetracycline. Bacterial titers were evaluated both by determining color changing units (CCU) and colony-forming units (CFUs) as previously described^51^. MICs were determined according to the Clinical & Laboratory Standards Institute guidelines^52^.

### Plasmid and *in vitro* methylation

The vector used for *M*. *hominis* transformation was the plasmid pMT85-Tet, which was derived from the Tn4001-based mini transposon plasmid pMT85^25^. The pMT85-Tet plasmid carried the tetracycline resistance gene *tet*(M), the expression of which was driven by the spiralin promotor (PS) instead of the gentamicin resistance gene^53,54^. Before transformation, the plasmid was methylated by the CpG methyltransferase from *Spiroplasma* sp. strain MQ1 (M. *Sss*I, New England Biolabs, Ipswich, Ma, United States) according to manufacturer’s recommendations.

### *M*. *hominis* transformation protocol

After determining the optimal conditions, the transformation protocol was performed as follows: a 10^8^ CFU/mL pre-culture was diluted from 10^−1^ to 10^−10^ and incubated for 24 h at 37 °C to obtain cultures at three different growth phases (e.g., early, mid-log and late log phase). The pool of these three different growth stages (which corresponded to the 10^−3^, 10^−4^ and 10^−5^ dilutions in three milliliters of culture) was centrifuged at 10,000 g, 4 °C for 20 min. The pellet was washed twice with cold T-Buffer (Tris 10 mM, pH 6.5) and centrifuged at 10,000 g, 4 °C for 10 min. After centrifugation, the cells were resuspended in 400 µL of cold 0.1 M CaCl_2_ and incubated at 4 °C for 30 min. Cold CaCl_2_-incubated cells (100 µL) were gently mixed with 10 µg of yeast tRNA (Life technologies, Carlsbad, CA, United States) and 10 µg of plasmid methylated with the methyltransferase SssI (New England Biolabs, Ipswich, Ma, United States). This mixture was aliquoted onto the surface of 1.5 mL of 40% PEG 8000 (Sigma-Aldrich, Saint-Louis, MO, United States) for 30 min. The contact was stopped by the addition of 7.5 mL of Hayflick arginine liquid medium and the cells were incubated for 3 hours at 37 °C. The transformation reaction was centrifuged at 8,000 g, room temperature for 10 min. The pellet was suspended in 1 mL of Hayflick arginine liquid medium and 200 µL was plated onto selective solid medium supplemented with 2 µg/mL of tetracycline (Sigma-Aldrich, Saint-Louis, MO, United States) and incubated at 37 °C with 5% CO_2_. Transformed *M*. *hominis* colonies appeared 3 to 7 days after transformation.

Colonies obtained on selective plates were picked and transferred into 1 mL of Hayflick arginine plus tetracycline (2 µg/mL) medium and incubated at 37 °C for 24 to 96 hours. Cultures were stocked at −80 °C. PCRs were performed with DNA extracts obtained using a NucleoSpin^®^ Tissue kit (Macherey-Nagel, Düren, Germany). After checking for the presence of the *tet*(M) gene and confirmation of the isolates as *M*. *hominis* by PCR (see below), the positive transformants were subcloned three times by successive passages on selective solid medium.

### Screening of *M*. *hominis* transformants by PCR

The *tet*(M) PCR mixture (50 µL final volume) contained 1X PCR buffer (Promega), 3 mM MgCl_2_, 0.2 mM dNTPs, 1 μM of each primers (IntMtet1 and IntMtet2), 1 U of HotStart G2 DNA polymerase (Promega), and 5 μL of DNA template. Amplification was performed as described by Dordet-Frisoni *et al*.^53^ Species determination was performed by amplification and sequencing (Eurofins genomics) of the 16S rRNA gene, according to^55^.

### Determination of the *tet*(M) gene insertion site by Single-Primer PCR

*M*. *hominis* total genomic DNA extractions were performed using 10 mL cultures with NucleoBond^®^ AXG20 columns and the NucleoBond^®^ Buffer Set III from Macherey Nagel according to the manufacturer’s instructions. Transposon insertion sites were then determined by single-primer PCR. The 25 µL final reaction volume contained 1X PCR Buffer (Promega), 3 mM MgCl_2_, 1 µM of SG9 primer (5′-TTTGGTTCAGAAACTGGTGCT-3′), 0.2 mM dNTPs, 0.1 µL of HotStart G2 DNA polymerase (Promega), and 2.5 µL of transformant DNA. The PCR amplification cycle was performed as previously described^53^. The insertion positions were determined by Sanger sequencing (Eurofins genomics) of the PCR products with the nested primer MT85-1 (5′-ACAGTAATTGCGGGTGGATC-3′).

### Analyses of mutant 28-2

The expression of the P75 gene (*Mhom132_02390*) in the *M*. *hominis* mutant 28-2 was assessed by semi-quantitative RT-PCR. Total RNA was extracted using a NucleoSpin^®^RNA plus kit (Macherey Nagel, Düren, Germany) according to manufacturer’s instructions. Reverse transcription was performed on total extracts with Avian Myeloblastosis Virus (AMV) Reverse Transcriptase following the recommendations provided by New England Biolabs. The presence of transcripts was finally verified by PCR amplification of cDNA. The 50 µL final reaction volume contained 1X PCR Buffer (Promega), 3 mM MgCl_2_, 1 µM of each primer, 0.2 mM dNTPs, 0.2 µL of HotStart G2 DNA polymerase (Promega), and 5 µL of cDNA. Two primer pairs were used, P75-F1 5′-GGCTTTTGGACTTTTAGCGC-3′ and P75-R1 5′-GGCTATTGTTTTCAGGGCTTG-3′, allowing the 5′ region of the gene to be amplified; and P75-F2 5′-GCAGCGCATGACGAATTAAG-3′ and P75-R2 5′-GCGCTTCATTTGGCTTGACT-3′, allowing the region around the transposon insertion site to be amplified. Amplification was performed as follows: 95 °C for 15 min, followed by 35 cycles of 1 min at 95 °C, 1 min at 58 °C and 1 min at 72 °C, with a final incubation for 5 min at 72 °C. The adhesion of the *M*. *hominis* mutant 28-2 to immobilized HeLa cells was assessed as previously reported^7^. A control with no HeLa cells was used to exclude the fact that *M*. *hominis* adheres to the plate (data not shown).

### Whole genome sequencing (WGS)

WGS with 1D Native barcoding genomic DNA (with EXP-NBD103 and SQK-LSK108) was performed using an Oxford Nanopore Technologies (ONT) GridION device by the Genome Transcriptome Facility of Bordeaux, France. Each step was performed according to the ONT recommendations. Each purified barcoded DNA sample was quantified using a Qubit fluorometer, and DNA sizes were checked using a Tapestation instrument. Sequencing was performed with a R9.4.1 Flowcell on the GridION device for 42 hours using live basecalling through the GridION dedicated basecaller Guppy. Whole genomes of the transformants 28-1 and 29-1 were analyzed to determine if multiple insertion of the *tet*(M) gene occurred. Genomes were assembled using a 500X coverage depth with reads having an average length of 8800 bp with Canu 1.7^56^ and was circularized with apc^57^.

## Supplementary information

Supplementary information

## Data Availability

All data analysed during this study are included in this published article. Additional datasets generated are available from the corresponding author upon request.
