# Supplementary material for: Random transposon insertion in the Mycoplasma hominis minimal genome
Source: Sci Rep. 2019 Sep 19;9:13554. doi: 10.1038/s41598-019-49919-y (PMC6753208; doi:10.1038/s41598-019-49919-y)
Supplement: Supplementary file 1 — Supplementary information [file 41598_2019_49919_MOESM1_ESM.docx]

**Supporting Information**

**Random transposon insertion in the *Mycoplasma hominis* minimal genome**

**Authors**

**Fabien Rideau^1-2^, Chloé Le Roy^1-2^, Eveline Sagné^3^, Hélène Renaudin^1-2^, Sabine Pereyre^1-2^, Birgit Henrich^4^, Emilie Dordet-Frisoni^3^, Christine Citti^3^, Carole Lartigue^5-6^**†***, Cécile Bébéar^1-2^**†**^*^**

^1^Univ. Bordeaux, USC-EA3671 Mycoplasmal and Chlamydial Infections in Humans, Bordeaux, France.

^2^INRA, USC-EA3671 Mycoplasmal and Chlamydial Infections in Humans, Bordeaux, France.

^3^IHAP, Université de Toulouse, INRA, ENVT, Toulouse, France.

^4^Institute of Medical Microbiology and Hospital Hygiene, Heinrich Heine University, Düsseldorf, Germany.

^5^INRA, UMR 1332 de Biologie du Fruit et Pathologie, F-33140 Villenave d'Ornon, France

^6^Univ. Bordeaux, UMR 1332 de Biologie du Fruit et Pathologie, F-33140 Villenave d'Ornon, France

†Co-last authors

*Corresponding authors: [cecile.bebear@u-bordeaux.fr](mailto:cecile.bebear@u-bordeaux.fr); carole.lartigue-prat@inra.fr

| System | Type I | | | Type II | | | | | | | Type III | Type IV |
| --- | --- | --- | --- | --- | --- | --- | --- | --- | --- | --- | --- | --- |
|  | EcoR124II | EcoKI | BcgIA | Sau96I | HaeIII | FokI | DpnII | Eco57I | BspRI | VspI |  | McrB-like |
| Specificity | 5'-GAAN_7_RTCG-3' | 5'-AACN_6_GTGC-3' | 5'-CGAN_6_TGC-3' | G/GNCC^a^ | GG/CC | GGATG(N)9/ | /GATC | CTGAAG (16/14) | GG/CC | AT/TAAT | ? | N4- and C5-methylcytosine |
|  |  |  |  |  |  |  |  |  |  |  |  |  |
| Adenine or Cytosine methylation | Adenine | Adenine | Adenine | Cytosine | Cytosine | Adenine | Adenine | Adenine 5 | Cytosine | Adenine 5 | Adenine? | - |
| Number of restriction sites in pMT85-Tet | 0 | 0 | 0 | 9 | 12 | 3 | 9 | 1 | 12 | 4 | ? | - |

**Table S1. Name and specificity of the R-M systems predicted in the *M. hominis* strains.**

^a^ Red letter indicates the methylation position.

**
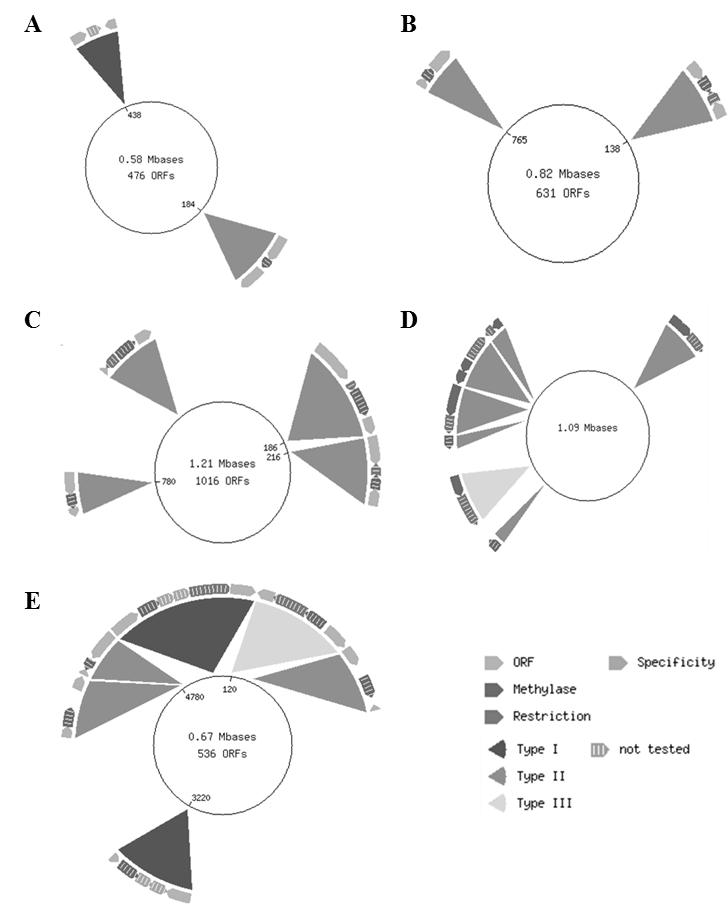
**

**Figure S1. Prediction of restriction-modification systems in different mycoplasma species by the Rebase database.** Representation of restriction-modification (R-M) systems of (A) *M. genitalium* G37, (B) *M. arthritidis* 158 L3-1, (C) *M. mycoides* subsp*. mycoides* PG1, (D) *M. mycoides* subsp*. capricolum* GM12 and (E) *M. hominis* PG21, based on the Rebase database. The smallest genome of *M. genitalium* G37 (A) only harbors one type I R-M system and one type II system. Cattle pathogens, such as *M. mycoides* subsp*. mycoides* strain PG1 (C) and *M. mycoides* subsp. *capri* strain GM12 (D), contain four type II R-M systems and seven R-M systems (six type II and one type III), respectively. *M. arthritidis* 158L3-1 (B) has only two type II R-M systems. Two type I, three type II and one type III systems are predicted to be present in the *M. hominis* PG21 genome by Rebase.


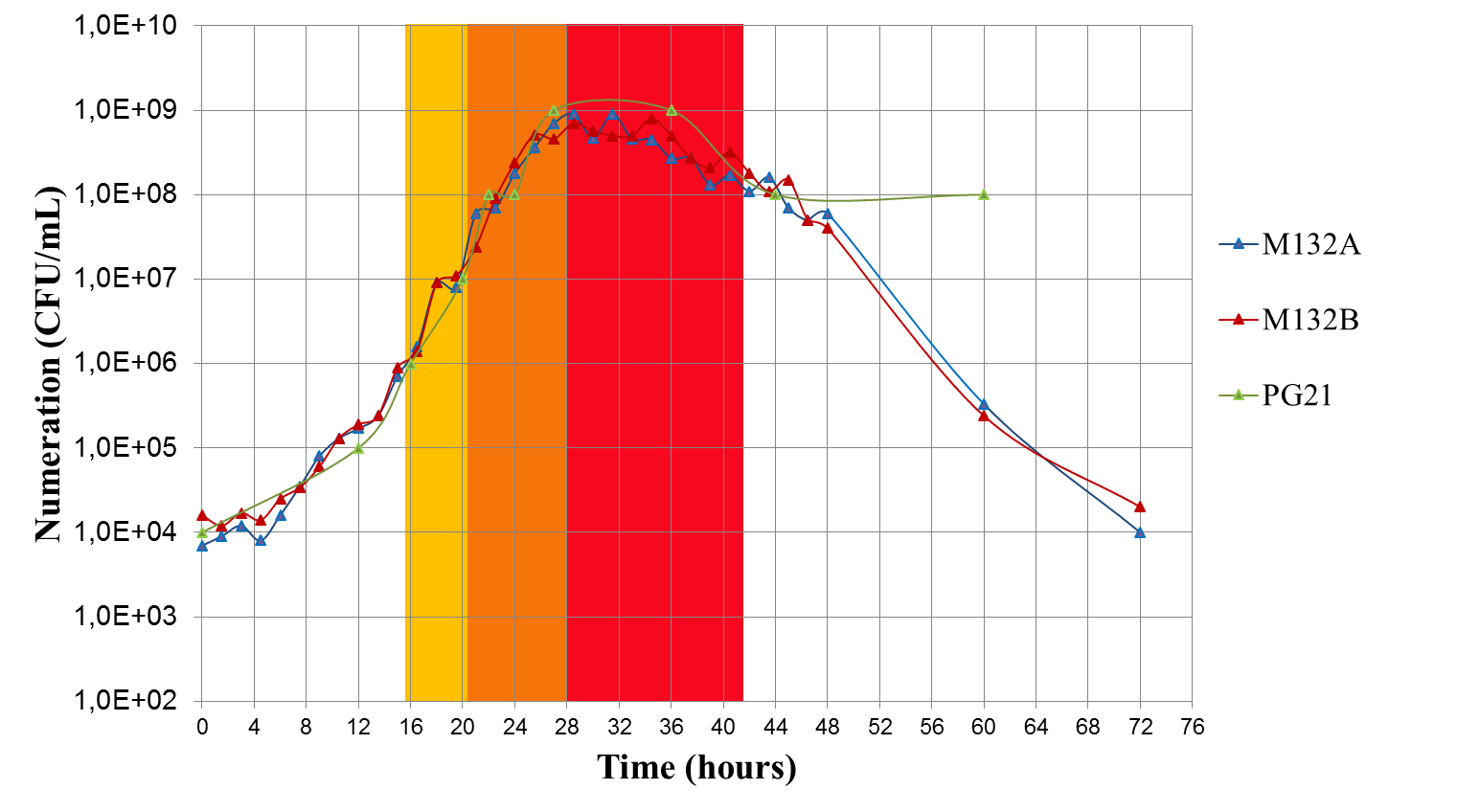


**Figure S2. *M. hominis* M132 growth curve.** Experiments were performed in duplicate (red and blue curves), and the *M. hominis* PG21 growth curve was added (Bébéar, personal data). Yellow, orange and red zones correspond to early, mid-log and late phases, respectively, that we defined. The doubling time for *M. hominis* strain M132 is approximately 65 minutes.


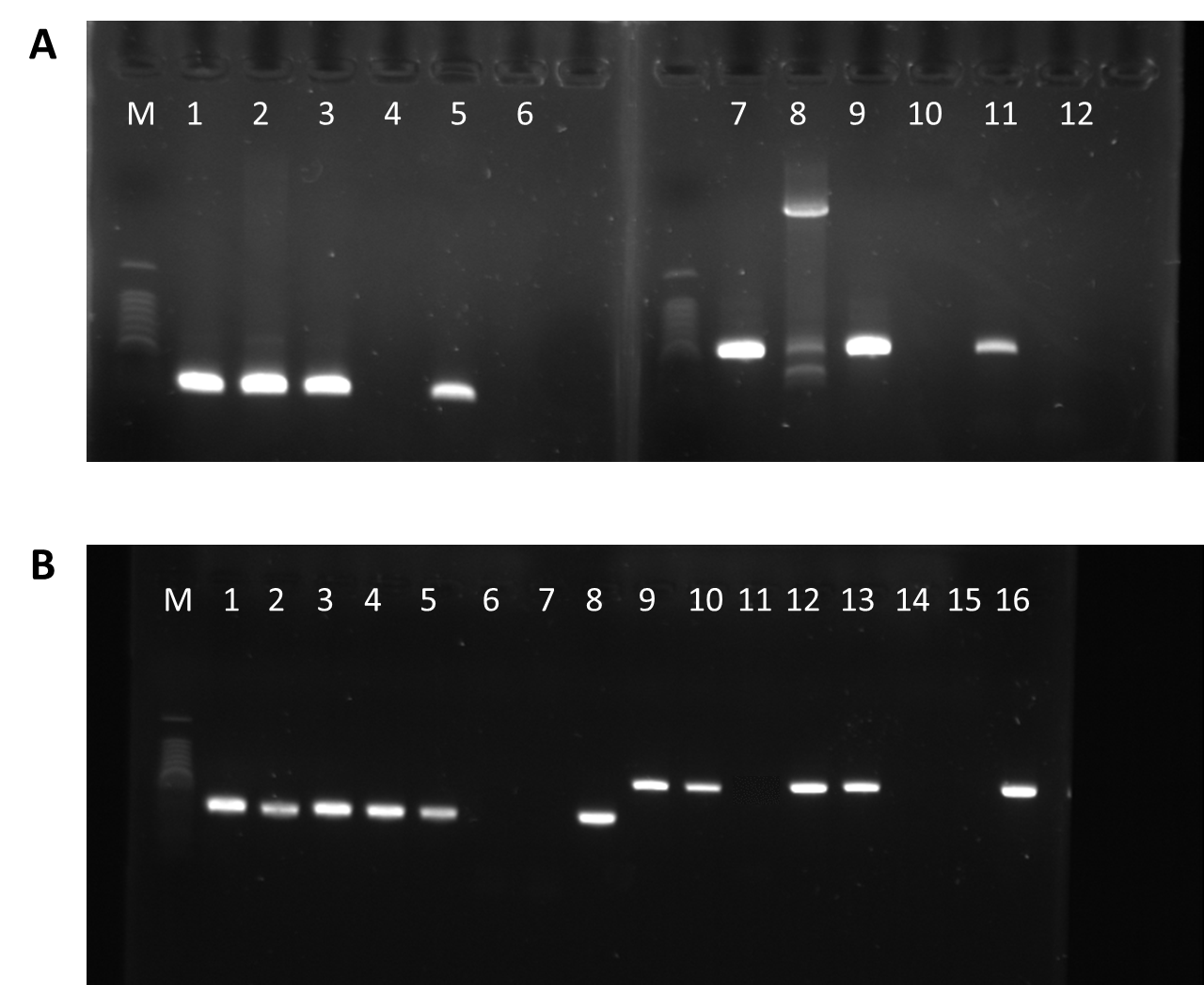


**Figure S3. Quantification of the P75 gene expression by semi-quantitative RT-PCR.** These gels were cropped to improve the presentation (Full-length gels are presented in supplemental figure S4).

(A) PCR amplification of the P75 gene. M: ladder 100 bp (Promega), 1: *M. hominis* M132, 2: mutant 28-2, 3: *M. hominis* PG21, 4: *M. arthritidis* 158L3-1, 5: mutant 39-5, 6: H_2_O, 7: *M. hominis* M132, 8: mutant 28-2, 9: *M. hominis* PG21, 10: *M. arthritidis* 158L3-1, 11: mutant 39-5, and 12: H_2_O. 1-6: primers P75-F1 and P75-R1, 7-12: primers P75-F2 and P75-R2.

(B) PCR amplification of cDNA after reverse transcription. 1: *M. hominis* M132 cDNA, 2: mutant 28-1, 3: mutant 28-2, 4: mutant 29-1, 5: mutant 39-5, 6: No RT control, 7: H_2_O, 8: *M. hominis* M132 DNA, 9: *M. hominis* M132 cDNA, 10: mutant 28-1, 11: mutant 28-2, 12: mutant 29-1, 13: mutant 39-5, 14: No RT control, 15: H_2_O, and 16: *M. hominis* M132 DNA. 1-8: primers P75-F1 and P75-R1, 9-16: primers P75-F2 and P75-R2.


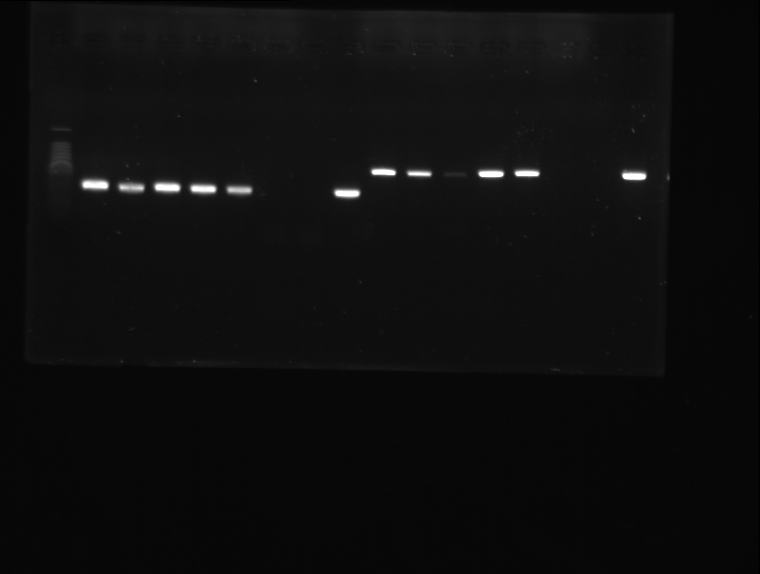

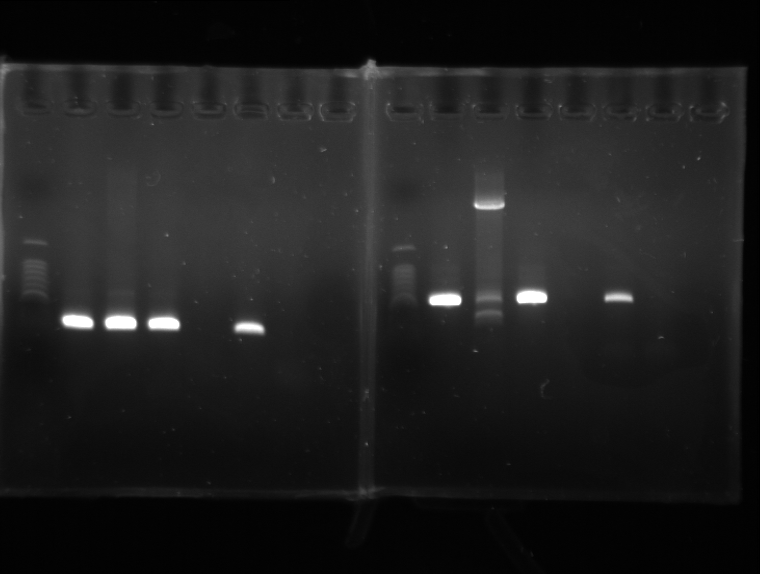


**Figure S4. Additional informations for figure S3. (A)** Full-length gel for figure S3 panel A. **(B)** Full-length gel for figure S3 panel B.

**B**

**A**
